# Comparison of the Gene Expression Profiles from Normal and Fgfrl1 Deficient Mouse Kidneys Reveals Downstream Targets of Fgfrl1 Signaling

**DOI:** 10.1371/journal.pone.0033457

**Published:** 2012-03-14

**Authors:** Simon D. Gerber, Ruth Amann, Stefan Wyder, Beat Trueb

**Affiliations:** 1 Department of Clinical Research, University of Bern, Bern, Switzerland; 2 Department of Rheumatology, University Hospital, Bern, Switzerland; Wayne State University, United States of America

## Abstract

Fgfrl1 (fibroblast growth factor receptor-like 1) is a transmembrane receptor that is essential for the development of the metanephric kidney. It is expressed in all nascent nephrogenic structures and in the ureteric bud. Fgfrl1 null mice fail to develop the metanephric kidneys. Mutant kidney rudiments show a dramatic reduction of ureteric branching and a lack of mesenchymal-to-epithelial transition. Here, we compared the expression profiles of wildtype and Fgfrl1 mutant kidneys to identify genes that act downstream of Fgfrl1 signaling during the early steps of nephron formation. We detected 56 differentially expressed transcripts with 2-fold or greater reduction, among them many genes involved in Fgf, Wnt, Bmp, Notch, and Six/Eya/Dach signaling. We validated the microarray data by qPCR and whole-mount in situ hybridization and showed the expression pattern of candidate genes in normal kidneys. Some of these genes might play an important role during early nephron formation. Our study should help to define the minimal set of genes that is required to form a functional nephron.

## Introduction

The mammalian kidney is a complex organ comprising thousands of nephrons that are connected by a branched collecting duct system [1]. The nephrons, the functional units of the kidney, filter the blood through a basement membrane and drain the filtrate via the collecting ducts to the bladder. In the mouse, nephron development is initiated at E10.5 when a caudal portion of the Wolffian duct near the hindlimbs bulges out and forms the ureteric bud. Signals from the metanephric mesenchyme induce the ureteric bud to branch in a stereotypical fashion to form the highly branched collecting duct system. The ureteric bud in turn releases signals that induce the metanephric mesenchyme to condense around the tips of the ureteric bud and to form the cap mesenchyme. Some cells of the cap mesenchyme undergo a mesenchymal-to-epithelial transition and develop into renal vesicles. These vesicles elongate, form s-shaped bodies and finally mature into nephrons.

Several signaling pathways are involved in the development of the nephron, including the Fgf/Fgfr, the Wnt/ß-catenin and the Notch/Presenilin pathway. To induce nephron formation, Wnt9b is secreted from the ureteric bud into the adjacent mesenchyme where it binds to Frizzled receptors and activates the canonical ß-catenin pathway [2], [3]. In response, the metanephric mesenchyme expresses the morphogens Fgf8 [4], [5] and Wnt4 [6]. Fgf8 is required for cell survival at different stages of nephrogenesis. Wnt4 induces cells from the cap mesenchyme to undergo the mesenchymal-to-epithelial transition, which finally leads to the formation of renal vesicles. Signaling by Notch and Presenilin is then needed to pattern the proximal tubule of the nephron [7], [8].

Recently, we have identified Fgfrl1 as a novel receptor that is essential for nephron development [9]. Fgfrl1 belongs to the Fgfr (fibroblast growth factor receptor) family of single transmembrane receptors (for review see [10]). Its extracellular domain resembles those of the classical Fgfrs in amino acid sequence and in that it contains three Ig-like loops. However, the intracellular domain differs from the classical receptors and does not possess any tyrosine kinase activity [11], [12]. The extracellular domain of Fgfrl1 interacts with heparin [13] and with Fgf ligands, primarily Fgf-2, -3, -4, -8, and -22 [14]. The intracellular domain binds to members of the Sprouty/Spred family that are known as negative regulators of the growth factor-mediated activation of the Ras/Raf/Erk signaling pathway [15]. During embryonic development, Fgfrl1 is expressed in tissues of the musculoskeletal system, including cartilage, bone and muscles [13], but also in the lung and the kidneys [9]. Information about the function of Fgfrl1 was gained from studies with mice, in which the Fgfrl1 gene was deleted by targeted inactivation [16], [17]. Fgfrl1 knock-out mice die shortly after birth due to malformation of the diaphragm. The mutant diaphragm muscle obviously is not strong enough to inflate the lungs after birth. However, the most striking phenotype of the Fgfrl1 deficient mice is the nearly complete absence of the metanephric kidneys. Utilizing organ cultures and different staining techniques, we demonstrated that Fgfrl1 deficiency leads to a dramatic reduction of ureteric branching and to a lack of mesenchymal-to-epithelial transition in the nephrogenic mesenchyme [9]. As a result, the mutant embryos lack any renal vesicles in their developing kidneys.

In the present study we used the DNA microarray profiling technique to identify genes that act downstream of Fgfrl1 signaling in the regulatory hierarchy of genes required for early nephron development. We confirmed reduced expression of Wnt4 and Fgf8 in the kidneys of the Fgfrl1 deficient mice. In addition, we identified more than 50 genes that are expressed at significantly reduced levels in our mutant mice. Many of these genes are involved in the Fgf/Fgfr, Wnt/ß-catenin, Bmp, Notch, and Six/Eya/Dach signaling pathway.

## Results

### Fgfrl1 is expressed throughout metanephric kidney development

**Figure 1:**
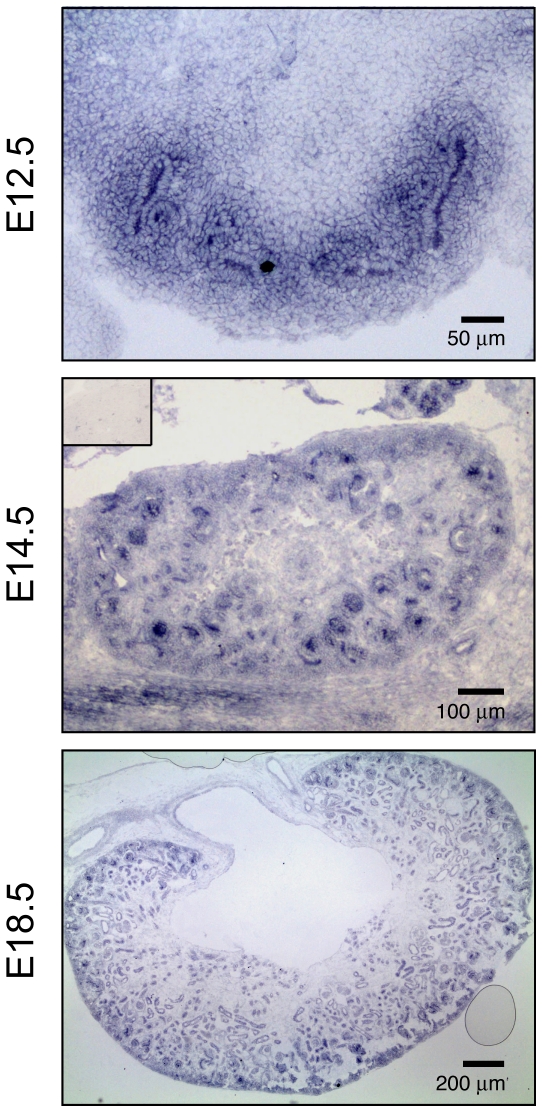
Expression of Fgfrl1 in the developing mouse kidney. Thin sections of embryonic mouse kidneys at E12.5, E14.5 and E18.5 were hybridized with a digoxigenin-labeled anti-sense RNA probe for Fgfrl1. After hybridization, the sections were incubated with alkaline phosphatase-conjugated antibodies against digoxigenin and the signal was developed with BM purple. Expression of Fgfrl1 was observed in the ureteric bud and in all nephrogenic structures. The inset of the panel at E14.5 shows a control section hybridized with the sense probe for Fgfrl1.

In a previous study we have used polyclonal antibodies on thin sections of E15.5 mouse kidneys to demonstrate that Fgfrl1 is expressed in nephrogenic structures of the cortical zone, with strong expression in renal vesicles, comma- and s-shaped bodies. Weaker staining was found in the undifferentiated mesenchyme and in the ureteric epithelium [9]. To verify these findings and to gain more information about Fgfrl1 expression during kidney development, we performed in situ hybridization on thin sections (SISH) at three developmental stages (Fig. 1). At E12.5, Fgfrl1 mRNA was highly expressed in the metanephric mesenchyme and in the ureteric bud. At E14.5, strong Fgfrl1 signal was detected in nascent nephrons and in the metanephric mesenchyme. At E18.5, Fgfrl1 signal was primarily found in tubules and nephrons. These results demonstrate that Fgfrl1 is expressed in developing nephrons and in the ureteric bud throughout kidney development, thus confirming our previous results obtained by immunohistochemistry.

### Transcriptional profiling of Fgfrl1 deficient kidneys

**Figure 2:**
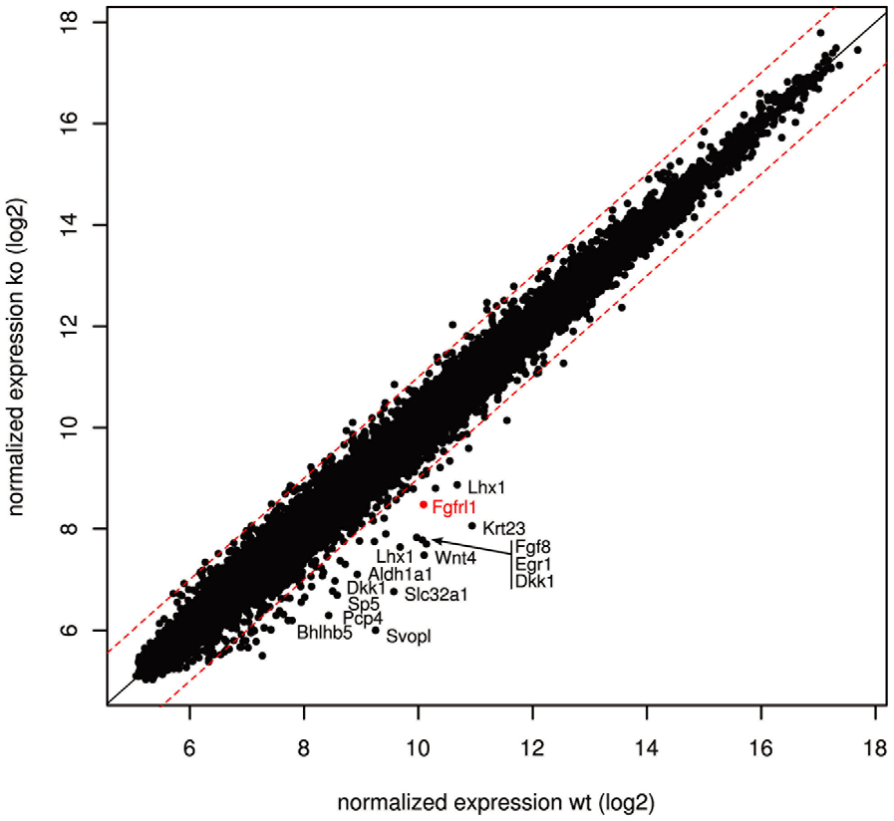
Identification of transcripts that are differentially expressed in Fgfrl1 deficient kidneys. The scatter plot shows average normalized signal intensities from three independent experiments using E12.5 kidneys from wildtype and Fgfrl1 knock-out mice. Each dot represents an individual gene. Dashed lines correspond to a fold change of 2. Transcripts that are down-regulated more than 3-fold are given by their gene symbol.

To determine how the absence of Fgfrl1 signaling would affect kidney development, we used Agilent DNA microarrays and compared mRNA levels between wildtype and Fgfrl1 deficient kidneys at E12.5. This time point was chosen because the first phenotypic differences between wildtype and Fgfrl1−/− kidneys are observed at E12.5 and because the first nephrogenic structures become visible at this developmental stage. Our efforts led to the identification of significant alterations in the transcriptome of the Fgfrl1 deficient kidneys. Of the ∼20,000 genes analyzed, 17 genes showed an up-regulation ≥2 fold and 56 genes showed a down-regulation ≥2 fold. None of the genes was up-regulated ≥3 fold and only 14 genes were down-regulated ≥3 fold (Fig. 2). Among the most strongly affected genes (indicated in the scatter plot of Fig. 2 by gene symbols) were Wnt4, Fgf8, Lhx1 and Fgfrl1, which had already been discovered as down-regulated markers in our previous study [9]. Expression of Eya1 and Six2, which define the uninduced mesenchyme, was barely affected by the absence of Fgfrl1 (fold change 1.3 and 1.0, respectively). Thus, the microarray data demonstrate the validity of our approach to identify mRNAs that may act downstream of Fgfrl1 in the regulatory hierarchy of genes required for nephron development.

Arbitrarily, we have chosen a minimal fold change >1.9 and a p-value cutoff <0.05 to generate a table of the most critical genes that were down-regulated in Fgfrl1 deficient kidneys (Table 1, for a list of all genes see GEO Series accession number GSE32013). This table included all the genes that had previously been found to be affected in the Fgfrl1 knock-out mouse (indicated in bold in Tables 1 and 2). Besides Wnt4, Pax8, Fgf8 and Lhx1, our list included 60 other genes that might contribute to the phenotype of the Fgfrl1 knock-out mice. Among these hits are many genes that have previously been described to be essential for normal kidney development, such as Spry1 [18], Etv4 [19] and Itga8 [20], but there are also several genes whose function in kidney development has not been appreciated so far, including Fzd10, Frzb, Il17rd and Dach1 (Table 1).

**Table 1:** Genes with reduced expression in Fgfrl1 deficient kidneys.

Nr	Gene Symbol	Gene Description	Gene ID	pValue	Fold Change	Verification Method		GUDMAP
1	Svopl	SV2 related protein homolog-like	320590	0.0022	9.5	qPCR		8950;7412
2	Krt23	keratin 23	94179	0.0014	7.4	qPCR,	WISH	
3	Slc32a1	solute carrier family 32 member 1	22348	0.0015	7.0	qPCR,	WISH	13958
**4**	**Wnt4**	**wingless-related MMTV integration site 4**	**22417**	**0.0013**	**6.2**	**qPCR,**	**WISH**	**8208;11295**
5	Dkk1	dickkopf homolog 1	13380	0.0106	5.4	qPCR,	WISH	9041
6	Egr1	early growth response 1	13653	0.0077	4.9	qPCR		6106;11301
7	Pcp4	immunoglobulin superfamily member 5	18546	0.0043	4.4	qPCR,	WISH	
**8**	**Fgf8**	**fibroblast growth factor 8**	**14179**	**0.0015**	**4.4**	**qPCR,**	**WISH**	
**9**	**Lhx1**	**LIM homeobox protein 1**	**16869**	**0.0028**	**4.1**	**qPCR,**	**WISH**	**5384;7928**
10	Sp5	trans-acting transcription factor 5	64406	0.0185	3.7		WISH	
11	Aldh1a1	aldehyde dehydrogenase family 1, subfamily A1	11668	0.0069	3.5	qPCR		
12	Clec18a	C-type lectin domain family 18 member A	353287	0.0055	3.4	qPCR,	WISH	
**13**	**Fgfrl1**	**fibroblast growth factor receptor-like 1**	**116701**	**0.0035**	**3.1**	**qPCR,**	**WISH**	
14	Bhlhb5	basic helix-loop-helix domain containing, class B5	59058	0.0002	3.0			5911
15	Hes5	hairy and enhancer of split 5	15208	0.0011	3.0			5928
16	Fzd10	frizzled homolog 10	93897	0.0180	2.9			8488
17	Gpx6	glutathione peroxidase 6	75512	0.0116	2.9			
18	Alx1	ALX homeobox 1	216285	0.0104	2.8			5336
19	Lmcd1	LIM and cysteine-rich domains 1	30937	0.0001	2.7			6340
20	Cck	cholecystokinin	12424	0.0247	2.7			
21	Amph	amphiphysin	218038	0.0158	2.7			
22	Aldh1a7	aldehyde dehydrogenase family 1, subfamily A2	26358	0.0164	2.6			
23	Galntl2	polypeptideN-acetylgalactosaminyltransferase-like 2	78754	0.0419	2.6			
24	Jag1	jagged 1	16449	0.0269	2.5	qPCR		8532;11379
25	Hey1	hairy/enhancer-of-split related with YRPW motif 1	15213	0.0101	2.4	qPCR		5912
26	Dll1	delta-like 1	13388	0.0162	2.4	qPCR		11371
27	Plekhg6	pleckstrin homology domain-containing family G6	213522	0.0065	2.4			
28	Akr1b7	aldo-keto reductase family 1, member B7	11997	0.0225	2.4			
29	Greb1	gene regulated by estrogen in breast cancer protein	268527	0.0017	2.3			8529;8891
30	Cxcr4	chemokine (C-X-C motif) receptor 4	12767	0.0233	2.3	qPCR		
31	Uncx	UNC homeobox	22255	0.0060	2.3	qPCR		5729
32	Notum	notum pectinacetylesterase homolog	77583	0.0061	2.3			
33	Bmp2k	BMP2 inducible kinase	140780	0.0434	2.3	qPCR		
34	C1qdc2	family with sequence similarity 132, member A	67389	0.0101	2.3			9273
35	Ism1	isthmin 1 homolog	319909	0.0106	2.3			
36	Lef1	lymphoid enhancer binding factor 1	16842	0.0071	2.3	qPCR		5539
37	Msx2	homeobox, msh-like 2	17702	0.0358	2.2	qPCR		5365
**38**	**Pax8**	**paired box gene 8**	**18510**	**0.0202**	**2.2**	**qPCR,**	**WISH**	**10742;11179**
39	Etv4	ets variant gene 4 (E1A enhancer binding protein)	18612	0.0414	2.2			5486;12534
40	Bmp2	bone morphogenetic protein 2	12156	0.0020	2.2	qPCR		8949
41	B3galt5	beta-1,3-galactosyltransferase 5	93961	0.0131	2.2			
42	Apom	apolipoprotein M	55938	0.0127	2.1			10784
43	Cxcl14	chemokine (C-X-C motif) ligand 14	57266	0.0175	2.1			8425
44	Ankrd56	mus musculus ankyrin repeat domain 56	78088	0.0165	2.1			
45	Frzb	frizzled-related protein	20378	0.0102	2.1	qPCR,	WISH	
46	Naaa	N-acylethanolamine acid amidase	67111	0.0077	2.1			
47	Osr2	odd-skipped related 2	107587	0.0094	2.0	qPCR		6335;13623
48	Il17rd	interleukin 17 receptor D	171463	0.0003	2.0	qPCR,	WISH	
49	Cpa2	carboxypeptidase A2, pancreatic	232680	0.0072	2.0			
50	Lbx2	ladybird homeobox homolog 2	16815	0.0400	2.0	qPCR		6590
51	Spry1	sprouty homolog 1	24063	0.0183	2.0		WISH	
52	Col13a1	collagen type XIII, alpha 1	12817	0.0034	2.0			8082
53	Dusp2	dual specificity phosphatase 2	13537	0.0003	2.0			
54	Rbm20	RNA binding motif protein 20	73713	0.0107	2.0			
55	Cdh4	cadherin 4	12561	0.0221	2.0			7763
56	Chrdl2	chordin-like 2	69121	0.0349	2.0			
57	Gldc	glycine decarboxylase	104174	0.0207	1.9			
58	Itga8	integrin alpha 8	241226	0.0062	1.9	qPCR		
59	Car4	carbonic anhydrase 4	12351	0.0006	1.9			
60	Metap2	methionine aminopeptidase 2	56307	0.0234	1.9			
61	Unc93a	unc-93 homolog A	381058	0.0076	1.9			
**62**	**Gdnf**	**glial cell line derived neurotrophic factor**	**14573**	**0.0445**	**1.9**	**qPCR**		
63	Dach1	dachshund 1	13134	0.0062	1.9	qPCR,	WISH	
64	Hs3st3b1	heparan sulfate(glucosamine)3-O-sulfotransferase3B1	54710	0.0342	1.9			11741

**Table 2:** Verification of differential gene expression by qPCR.

Gene	Fold change wt/ko qPCR				Fold change wt/ko
	E11.5	E12.5	E14.5	E16.5	array E12.5
Slc32a1	0.8	>20	13.4	11.7	7
Krt23	7.5	17.4	1.5	3.6	7.4
Pcp4	2.7	17	6	1	4.4
**Wnt4**	**2.3**	**13.3**	**3.8**	**2.3**	**6.2**
**Pax8**	**1.5**	**9.6**	**3.9**	**4.7**	**2.2**
Svopl	11.1	9.4	10.6	>20	9.5
**Fgf8**	**3.2**	**9.2**	**16.4**	**>20**	**4.4**
Il17rd	2.3	8.5	5.9	4.9	2
Lef1	2.4	8.2	0.8	2.1	2.3
**Lhx1**	**0.7**	**7.7**	**12.7**	**>20**	**4.1**
Jag1	2	7.4	5.7	3.9	2.5
Uncx	1.6	7	10.1	17.2	2.3
Itga8	3.1	6.9	2.6	2.1	1.9
**Fgfrl1**	**>20**	**6.8**	**7.5**	**>20**	**3**
Frzb	2.5	6.1	2.9	3.4	2.1
Dach1	1.7	6	4	5.1	1.9
Bmp2	0.8	5.2	7.9	6.3	2.2
Osr2	2.9	4.8	11.7	>20	2
Egr1	1	4.6	2.1	1.5	4.9
Aldh1a1	9.8	4	6.3	3.7	3.5
**Gdnf**	**3.5**	**3**	**3.1**	**4.1**	**1.9**
Cxcr4	1.7	3.4	3.7	3.1	2.3
Dkk1	2.5	3	13.1	>20	5.4
Clec18a	2.1	2.3	>20	>20	3.4
Lbx2	0.8	2.1	2.1	1.5	2
Bmp2k	1	2	1	1	2.3
Dll1	1.1	1.7	7.4	13.6	2.4
Msx2	0.4	1.6	1.2	0.7	2.2
Hey1	1.6	1.4	3.3	5	2.4
Tcfcp2l1	3.1	1.4	1.2	3.6	0.5
Cxcl12	2.2	1.3	0.7	1.2	0.5
Rps9	1.9	1.2	1.8	2.4	1.1
Gapdh	1	1	1	1	1
Col1a1	0.8	1	0.7	0.5	0.5

### Validation by quantitative PCR

In order to confirm the results of the DNA microarray, we quantified the mRNA levels of selected genes by RT-PCR. For this purpose, we focused on hits that had yielded large differences between wildtype and mutant kidneys. In this way, 29 down-regulated genes, two control genes (Rps9 and Gapdh) and 3 up-regulated genes were analyzed by qPCR (Table 2). For the majority of the down-regulated hits, the qPCR results were found to be in good agreement with the microarray data, although the differences (fold changes) observed by qPCR were larger than those determined by microarray analysis. Only three genes (Dll1, Msx2, Hey1) showed a minimal fold change <1.9 by qPCR, which had been selected as cutoff above.

In sharp contrast to the down-regulated transcripts, none of the up-regulated transcripts (Tcfcp2l1, Cxcl12, Col1a1) could be confirmed by qPCR as the fold changes observed by qPCR were between 1.0 and 1.4. We may therefore conclude that the lack of Fgfrl1 expression in the mutant kidneys was barely compensated for by the up-regulation of other genes. In particular, the classical receptors Fgfr1-Fgfr4 did not show altered expression in the mutant kidneys.

For the microarray experiment, we had used E12.5 kidneys because overt differences in the phenotype between wildtype and Fgfrl1 deficient kidneys appear at this developmental stage. However, changes in gene expression may occur prior to the actual appearance of an altered phenotype and these changes may or may not persist throughout kidney development. We therefore analyzed the 29 down-regulated genes, the 3 up-regulated genes and the two control genes also at three additional time points during kidney development, one prior to and two after the stage used for the microarray experiment (E11.5, E14.5, E16.5). These data are also included in Table 2. Although some of the results might be difficult to interpret, the majority of the genes can clearly be grouped into two different categories. One category includes genes whose fold changes show a bell-shaped curve during embryonic development (e.g. Slc32a1, Pcp4, Wnt4, Lef1, Itga8, Egr1). These genes might preferentially be required during the initial stages of nephron development. The other category includes genes whose fold changes steadily increase throughout development (e.g. Fgf8, Lhx1, Uncx, Osr2, Dkk1, Clec18a, Dll1). It is likely that these genes are particularly needed at later stages of nephron development.

### Validation by WISH

**Figure 3:**
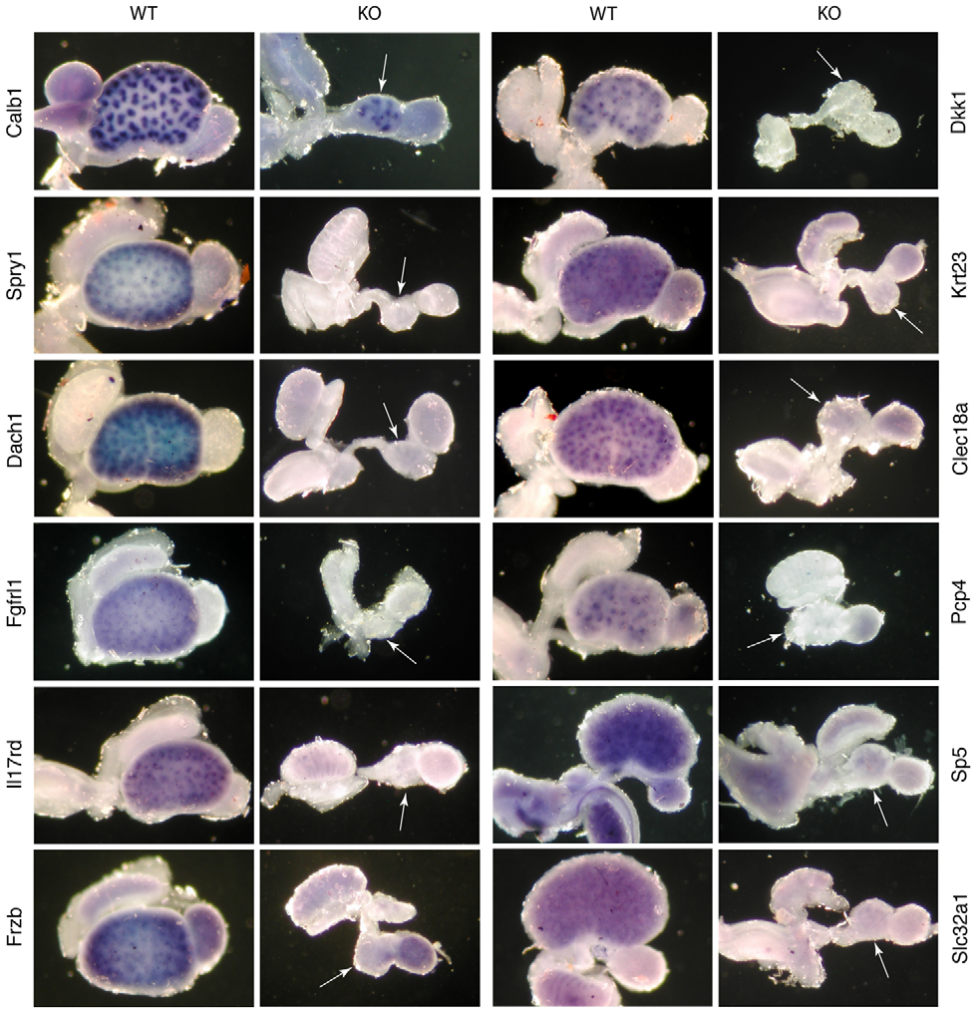
Whole mount in situ hybridization (WISH) of selected marker genes in E14.5 kidneys. Kidneys from wildtype and Fgfrl1 knock-out mice were hybridized with digoxigenin-labeled RNA probes and the probes were detected with alkaline phosphatase conjugated antibodies against digoxigenin. All probes produced clear expression patterns in the wildtype kidneys. In contrast, the same probes did not display any pattern in knock-out kidneys, with the exception of Calb1 that was included as a positive control. The white arrows indicate the kidney rudiments of the knock-out embryos.

To further validate our data and to examine the spatial expression pattern of the genes in the kidney, we performed whole-mount in situ hybridization experiments (WISH). For this purpose, we used E14.5 kidneys because samples at this stage revealed a clearer expression pattern than samples at E12.5 due to the increased size and the generally stronger gene expression level [9]. We primarily focused on genes, which had shown a difference >3 and which had not yet been annotated in the GUDMAP database (www.gudmap.org). In total 12 genes were tested, including the positive control Calb1 (Fig. 3). Calb1 revealed the expected expression pattern in the ureteric bud and its derivatives, in both the wildtype and the Fgfrl1 knock-out kidneys. In contrast, expression of the other 11 genes was significantly reduced or even absent in the Fgfrl1 deficient kidneys (Fig. 3). Likewise, expression of the four nephrogenic marker genes Wnt4, Fgf8, Lhx1 and Pax8 that had been analyzed by WISH in our previous study was not detected in the Fgfrl1−/− kidneys [9]. Thus, our in situ hybridization data confirmed the microarray and the qPCR results.

It is of interest to note that most of the genes detected by WISH in the wildtype kidneys were expressed in the ureteric bud and/or in developing nephrogenic structures, such as renal vesicles and s-shaped bodies. These genes included Wnt4, Fgf8, Lhx1 and Pax8 from the previous study and Spry1, Dach1, Dkk1, Il17rd, Frzb, Krt23, Clec18a and Pcp4 from the present study (Fig. 3). In contrast, expression of the transcription factor Sp5 and the amino acid transporter Slc32a1 was rather diffuse, revealing no distinct pattern. We may therefore conclude that the majority of the differentially expressed genes identified by microarray analysis and confirmed by WISH showed expression in renal vesicles and s-shaped bodies, structures that are missing in our Fgfrl1 deficient mice.

## Discussion

In the present study we demonstrated that Fgfrl1 is expressed in the ureteric bud and in all nephrogenic structures of wildtype mice, including renal vesicles and comma- and s-shaped bodies. Mice lacking the Fgfrl1 gene do not develop any nephrogenic structures. A careful analysis of the kidney transcriptome from wildtype and knock-out mice allowed us to identify more than 50 genes that act - directly or indirectly - downstream of Fgfrl1 in the regulatory cascade of genes required for early nephron development. Many of these genes appear to be involved in well-established signaling pathways. However, it cannot be deduced from our study whether all these genes are directly involved in the same signaling pathway as Fgfrl1. Since Fgfrl1 deficient mice do not develop any renal vesicles it is also possible that some of the identified mRNAs lack in the Fgfrl1 null mice simply because they are normal constituents of renal vesicles. To minimize such “secondary” effects, we analyzed kidneys at stage E12.5 where the first nephrogenic structures become visible and where the first phenotypic differences between wildtype and Fgfrl1−/− mice are observed.

### FGF signaling pathway

By virtue of our microarray approach, we found five down-regulated genes (Fgf8, Spry1, Il17rd, Ism1, Etv4) that are involved, directly or indirectly, in Fgf signaling. Fgf8 is essential for nephron formation as mice lacking this gene do not progress beyond the renal vesicle stage [4], [5]. Interestingly, Fgf8 is one of the best ligands for Fgfrl1 as demonstrated by a ligand dot blot assay [14]. Spry1 is an antagonist of Fgf signaling, which is crucial for normal outgrowth of the ureteric bud as Spry1−/− embryos possess supernumerous ureteric buds [18]. Il17rd (also termed Sef), Ism1 and Etv4 belong to the Fgf synexpression group [20]–[22]. This group comprises several genes that show a similar spatiotemporal expression pattern and that may serve a similar function during development. Fgfrl1 [10], Il17rd [21], Spry2 and Spry4 [23] have been reported to act as negative regulators of Fgf signaling. The exact function of Fgfrl1 is not yet known, but we have speculated that it might act as a decoy receptor, which sequesters Fgf ligands away from the actively signaling receptors, or as a dominant negative binding partner, which interacts with the other receptors and inhibits transphosphorylation of the intracellular domains [13]. If this were true, one would expect up-regulation of genes that act downstream of FGF signaling, such as Fgfr1, Fgfr2 or FGF8, resulting in increased numbers of ureteric buds and nephrogenic structures. In sharp contrast, Fgfrl1 null mice have a phenotype with renal dysplasia [9] very similar to mice with a conditional disruption of Fgf8 [4], [5] or a compound disruption of the two receptors Fgfr1 and Fgfr2 [24]. This observation suggests that Fgfrl1 might act as a positive regulator of FGF signaling during kidney development and not as a decoy receptor.

### WNT signaling pathway

The Wnt signaling pathway is often activated in concert with Fgf signaling during developmental processes [25]. We found by our microarray approach at least five genes that have been implicated in Wnt signaling, namely Wnt4, Fzd10, Frzb, Lef1 and Sp5. Among these hits, Wnt4 ranked at the top with a 6-fold expression difference between wildtype and knock-out mice when measured by microarray and 13-fold when verified by qPCR experiments. Wnt4 is usually expressed in the metanephric mesenchyme, where it induces the mesenchymal-to-epithelial transition. Therefore mice lacking Wnt4 activity show a greatly reduced number of nascent nephrogenic structures [6].

In a recent study, Valerius & McMahon [26] performed a transcriptional profiling screen using Wnt4 deficient kidneys. The authors identified 236 genes with differential expression levels between wildtype and Wnt4−/− kidneys. Interestingly, several genes exhibiting reduced expression in their study were also found to be down-regulated in our study, such as Dll1, Pcp4, Dkk1, Pax8, Fgf8, Lhx1, Hes5, Hey1 and Egr1. This result can be explained by the fact that Fgfrl1 acts upstream of Wnt4 in the cascade of regulatory genes as Fgfrl1 deficient mice lack Wnt4 expression in their kidney rudiments.

The other members of the Wnt signaling cascade that were significantly down-regulated in our study appear to have diverse functions. Fzd10 is one of the receptors for Wnt ligands. Frzb is a secreted, frizzled-related receptor that interferes with Wnt signaling. Lef1 is a transcription factor participating in Wnt signaling. Canonical Wnt signaling leads to the stabilization of ß-catenin, which - after translocation to the nucleus - interacts with transcription factors of the Lef/Tcf family to induce expression of target genes [27]. Sp5 is a member of the Sp1 transcription factor family. It ranks among the ten best hits of our microarray analysis. Fujimura et al. [28] presented evidence that Sp5 is involved in Wnt signaling since constitutive activation of the Wnt/ß-catenin pathway resulted in the up-regulation of Sp5 expression in the mouse telencephalon.

### Bmp signaling

With our microarray, we found reduced expression of Bmp2 and Bmp2k. The Bmps play a key role in the development of the skeleton, but they are also involved in patterning of the metanephric kidney [29]. Bmp2 is expressed in the distal renal vesicle as shown by Georgas et al. [30]. The authors suggested that Bmp2 might be involved - together with other factors - in the fusion of the renal vesicle with the ureteric tip. Bmp2k is a serine/threonine protein kinase whose expression is induced after addition of Bmp2 to prechondroblastic cells in order to trigger their differentiation [31]. However, the role of Bmp2k during nephron formation has not yet been investigated.

### Notch signaling pathway

Notch signaling is required to pattern the distal and proximal tubule of the nephron [32]. In our microarray, the Notch ligands Jag1 and Dll1 as well as the downstream effector Hey1 were down-regulated. However, Hey1 and Dll1 showed only mild reduction when validated by qPCR (1.4- and 1.7-fold, respectively), in contrast to Jag1, whose expression was reduced 7.4-fold. Jag1 is known to interact with Notch2 and other Notch receptors [33]. It regulates ureteric budding and branching by crosstalk with Gdnf/Ret signaling [34]. It is therefore likely that downregulation of Jag1 in the Fgfrl1 deficient kidneys reduces Notch signaling and hence interferes with normal nephron development.

### Six-Eya-Dach signaling pathway

Six (sine oculis), Eya (eyes absent) and Dach are transcription factors that constitute the “retinal determination network”, whose loss inhibits eye development and whose forced expression leads to ectopic eye formation [35]. It is believed that the three factors form a complex that binds to the promoter region of target genes to control eye formation in mammals and insects. Besides their function in eye formation, Six1 and Eya1 are also involved in the development of the metanephric kidneys of mammals [36] as targeted deletion of either one of these genes leads to severe kidney malformation. Targeted disruption of Dach1, however, does not seem to have any obvious effect on kidney or eye formation, although mice without Dach1 exhibit postnatal lethality [37].

In our gene array, Six1 and Eya1 were not differentially expressed (fold change 1.1 and 1.3, respectively). This is in contrast to Dach1, which was significantly down-regulated (qPCR 6.0-fold, Table 2). It is therefore likely that Six1 and Eya1 act upstream, while Dach1 acts downstream of Fgfrl1 in the same regulatory network of genes that are required for kidney development. As a matter of fact, the phenotypes of Fgfrl1, Eya1 and Six1 deficient mice look intriguingly similar. All of them show kidney and bone malformations [9], [38], [39]. In addition, Six1 and Fgfrl1 knock-out mice exhibit defects in the diaphragm [16], [40]. Dach1 is expressed in developing nephrons, primarily in comma- and s-shaped bodies [41]. This is consistent with our WISH experiments where a prominent signal for Dach1 was found in the metanephric mesenchyme surrounding the ureteric tips (Fig. 3). Brunskill et al. [42] observed strong upregulation of Dach1 during nephron formation, especially when the stage of the renal vesicle was compared with that of the s-shaped body (46-fold). It is likely that Fgfrl1 is involved in this upregulation since our Fgfrl1 deficient kidneys show strongly reduced Dach1 expression.

### Conclusions

We have identified a number of genes that act downstream of Fgfrl1 signaling in the regulatory hierarchy of genes required for early nephron development. Several of these genes are involved in Fgf/Fgfr, Wnt/ß-catenin, Bmp, Notch, and Six/Eya/Dach signaling. The downregulation of these genes might be responsible for the lack of nephrogenesis observed in Fgfrl1 knock-out mice. For some of the identified genes, a potential involvement in the development of the metanephric kidneys has not yet been appreciated (e.g. Fzd10, Frzb, Il17rd and Dach1). Our study should therefore help to define the minimal set of genes that is required for normal nephron formation.

## Materials and Methods

### Animals

All animal work was conducted according to the relevant national guidelines and was approved by the Amt für Landwirtschaft und Natur of Bern (approval number 69/09). The Fgfrl1 deficient mice have previously been described [9]. Littermates of wildtype and Fgfrl1 knock-out mice were used for all experiments. For an exactly timed pregnancy, the noon of the day, at which a vaginal plug was detected, was considered as E0.5.

### DNA Microarray analysis

Kidney rudiments were dissected in parallel from wildtype and Fgfrl1 knock-out mice of stage E12.5. Total RNA was extracted from pooled kidney rudiments (n = 6–9) with the GeneElute miniprep kit from Sigma-Aldrich Co. The quality of the RNA was assessed with an Agilent 2100 Bioanalyser (Agilent Technologies, Palo Alto, CA, USA). Three individual RNA preparations from wildtype mice and three individual RNA preparations from Fgfrl1 knock-out mice were separately transcribed into double stranded cDNA in the presence of RNA poly-A controls (RNA Spike-In Kit, Agilent 5188-5279). The cDNAs were transcribed with T7 polymerase into cRNA utilizing cyanine 3-CTP (Cy3) for knock-out and cyanine 5-CTP (Cy5) for wildtype samples, respectively (Agilent 5190-0444). No amplification step was performed. The three pairs of fluorescently labeled cRNA were hybridized for 17 h at 65°C to an Agilent gene expression array (Whole Mouse Genome Microarray 4×44 K, G4122F) according to the instructions of the manufacturer utilizing reagents from the Gene Expression Hybridization Kit (Agilent 5188-5242). The slides were washed and scanned using an Agilent G2565BA microarray scanner. Signals were extracted from images using the Agilent Feature Extraction software version 10. Data analysis was performed on the R platform for statistical computing with packages from the Bioconductor project [43]. Gene annotation and identifier conversions were retrieved from the Mouse Genome Database (MGD, http://www.informatics.jax.org). All microarray data were deposited in the GEO database and comply with MIAME standards (accession number GSE32013).

### Quantitative PCR analysis

Quantitative PCR was performed as previously described [9]. In brief, the RNA was transcribed into first strand cDNA with reverse transcriptase from Moloney Murine Leukemia Virus. The cDNAs were quantified by real time PCR on an ABI 7500 platform using the primer pairs listed in Table S1.

### In situ hybridization

Whole-mount in situ hybridization (WISH) was performed as described before [9]. Different hybridization probes were generated by PCR utilizing cDNA prepared from E16.5 kidneys and the primer pairs listed in Table S2.

In situ hybridization on thin sections (SISH) was performed according to Koch et al. [44] with minor modifications. Sense and anti-sense riboprobes for Fgfrl1 were prepared by reverse transcription from the full-length cDNA cloned into the vector pcDNA3.1 (+/−) using the digoxigenin RNA labeling kit from Roche Diagnostics (Rotkreuz, Switzerland). Dissected kidneys were fixed with paraformaldehyde (4% PFA in PBS, overnight at 4°C) and incubated in a sucrose solution (30% sucrose in PBS, overnight at 4°C). Equilibrated samples were embedded in Tissue-Tek, frozen on dry ice and cut to 12 µm sections. Cryosections were fixed with PFA (4% in PBS, 20 min at RT) and digested with proteinase K (10 µg/ml, 10 min at RT). After refixation (4% PFA, 20 min), sections were acetylated (0.25% acetic anhydride, 0.1 M triethanolamine, pH 8.0, 10 min). Acetylated sections were prehybridized in hybridization buffer (50% formamide, 4× SSC, 2× Denhardt's solution, 5% dextran sulfate, 100 µg/ml yeast tRNA, 5 h at RT) and hybridized with the Fgfrl1 probe (overnight at 68°C with 1 µg/ml of the digoxigenin-labeled riboprobe). After a series of washing steps (wash 1: 0.2× SSC, 30 min at 60°C; wash 2: 50% formamide, 2× SSC, 30 min at RT; wash 3: 0.2× SSC, 10 min at RT; wash 4: 0.1 M maleic acid, 0.15 M NaCl, 0.1% Tween-20, pH 7.4, 15 min at RT) the sections were blocked with BSA (3% in Tris buffered saline, 2 h at RT) and incubated with anti-digoxigenin antibodies (alkaline phosphatase-conjugated Fab fragments from Roche, diluted 1∶2000, overnight at 4°C). The slides were rinsed and equilibrated with detection buffer (0.1 M Tris, 0.1 M NaCl, 50 mM MgCl_2_, pH 9.5). Finally, the hybridization signal was developed with BM Purple (Roche, 12–24 h at RT) and the slides were photographed under an Olympus BX-51 microscope.

## Supporting Information

Table S1 **Primers used for RT-PCR.**
(DOC)

Table S2 **Primers used for WISH. Underlined nucleotides indicate restriction sites used for subcloning.**
(DOC)
