# Supplementary material for: Comparison of the Gene Expression Profiles from Normal and Fgfrl1 Deficient Mouse Kidneys Reveals Downstream Targets of Fgfrl1 Signaling
Source: PLoS One. 2012 Mar 14;7(3):e33457. doi: 10.1371/journal.pone.0033457 (PMC3303837; doi:10.1371/journal.pone.0033457)
Supplement: Table S1 — Primers used for RT-PCR. (DOC) [file pone.0033457.s001.doc]

| **Table S1. Primers used for RT-PCR** | |  |
| --- | --- | --- |
| Primer name | **Sequence (5'→3')** | Accession number |
| Aldh1a1 up | GAAAGAAGGAGCCAAACTGGA | [NM_013467](http://www.ncbi.nlm.nih.gov/nuccore/NM_013467.3) |
| Aldh1a1 low | TGCACTGGTCCAAATATCTCC |  |
| Bmp2 up | CGGACTGCGGTCTCCTAA | [NM_007553](http://www.ncbi.nlm.nih.gov/nuccore/NM_007553.2) |
| Bmp2 low | GTTCCTCCACGGCTTCTTC |  |
| Bmp2k up | CAGCAACCACAAACCCAAA | [NM_080708](http://www.ncbi.nlm.nih.gov/nuccore/NM_080708.1) |
| Bmp2k low | AATCTCCAGTCTCCCTGCTG |  |
| Chl1 up | CATCTCAACCCAAGGAAATGA | NM_007697 |
| Chl1 low | ACTATTTCTTCTTCCCACTCTTCG |  |
| Clec18a up | CTGAAGGGCTGCAGTACAGA | [NM_181549](http://www.ncbi.nlm.nih.gov/nuccore/NM_181549.3) |
| Clec18a low | TTTATATCCCAGTTGCCTCCA |  |
| Col1a1 up | CCGGCTCCTGCTCCTCTTAG | NM_007742 |
| Col1a1 low | CCATTGTGGCAGATACAGATCAAG |  |
| Cxcl12 up | GGTCCACCTCGGTGTCCT | NM_001012477 |
| Cxcl12 low | CAGGCTGACTGGTTTACCG |  |
| Cxcr4 up | TGGAACCGATCAGTGTGAGT | [NM_009911](http://www.ncbi.nlm.nih.gov/nuccore/NM_009911.3) |
| Cxcr4 low | CCGACTATGCCAGTCAAGAAG |  |
| Dach1 up | GTGTGCAATGTGGAACAGGT | [NM_007826](http://www.ncbi.nlm.nih.gov/nuccore/NM_007826.2) |
| Dach1 low | TTAGGAGGCCTTCCAGGTC |  |
| Dkk1 up | GCGGGAACAAGTACCAGACT | [NM_010051](http://www.ncbi.nlm.nih.gov/nuccore/NM_010051.3) |
| Dkk1 low | GACGGAGCCTTCTTGTCCTT |  |
| Dll1 up | GAAGGTTGCTCTGTGTTCTGC | [NM_007865](http://www.ncbi.nlm.nih.gov/nuccore/NM_007865.3) |
| Dll1 low | CACACCCTGGCAGACAGAT |  |
| Egr1 up | CCTATGAGCACCTGACCACAGAG | [NM_007913](http://www.ncbi.nlm.nih.gov/nuccore/NM_007913.5) |
| Egr1 low | GTCGGAGGATTGGTCATGCTC |  |
| Frzb up | GGGACACCGTCAATCTTTATACC | [NM_011356](http://www.ncbi.nlm.nih.gov/nuccore/NM_011356.4) |
| Frzb low | ATTAGAGTTCCTGCCAGACTTCTG |  |
| Gapdh up | AGGTCGGTGTGAACGGATTTG | [NM_008084](http://www.ncbi.nlm.nih.gov/nuccore/NM_008084.2) |
| Gapdh low | GCGGAGATGATGACCCTTTTG |  |
| Hey1 up | GCGTGAGTGGGATCAGTGT | [NM_010423](http://www.ncbi.nlm.nih.gov/nuccore/NM_010423.2) |
| Hey1 low | CTTCTCGATGATGCCTCTCC |  |
| Il17rd up | GGCAGTCACCATTCTTTGGT | [NM_134437](http://www.ncbi.nlm.nih.gov/nuccore/NM_134437.3) |
| Il17rd low | AGAATCAGCTGTTGGCACTGT |  |
| Itga8 up | CCTGAGATCAACCCACAGG | [NM_001001309](http://www.ncbi.nlm.nih.gov/nuccore/NM_001001309.2) |
| Itga8 low | TCGATCTGACCTTTAGGACTGC |  |
| Jag1 up | CCTCAGGCTTTGAGTGTGAGT | [NM_013822](http://www.ncbi.nlm.nih.gov/nuccore/NM_013822.4) |
| Jag1 low | GCCAAGGCAGTCATTGATATTTA |  |
| Krt23 up | GGTGGCATCAGGAGAGAGAA | [NM_033373](http://www.ncbi.nlm.nih.gov/nuccore/NM_033373.1) |
| Krt23 low | GGCCATCTTACCGTCCACTA |  |
| Lbx2 up | GTCCCCGAAGCACCTTCT | [NM_010692](http://www.ncbi.nlm.nih.gov/nuccore/NM_010692.3) |
| Lbx2 low | CCGGGGCGGCTCTGCCTT |  |
| Lef1 up | CGGACAGTGACCTAATGCAC | [NM_010703](http://www.ncbi.nlm.nih.gov/nuccore/NM_010703.3) |
| Lef1 low | TCTCTCTTTCCGTGCTAGTTCA |  |
| Msx2 up | GGAACGCAGGGTCAAGGTC | [NM_013601](http://www.ncbi.nlm.nih.gov/nuccore/NM_013601.2) |
| Msx2 low | GTGGTGGGGCTCATATGTCTG |  |
| Osr2 up | CCAGGCAGACATCGGTTC | [NM_054049](http://www.ncbi.nlm.nih.gov/nuccore/NM_054049.2) |
| Osr2 low | TGCTCCCCATCTTCCTGA |  |
| Pcp4 up | CACCCAGCAGCTCTTTTTAAG | [NM_008791](http://www.ncbi.nlm.nih.gov/nuccore/NM_008791.2) |
| Pcp4 low | GCTCCGGCACTTTGTCTCT |  |
| Rps9 up | GATCCCCGGCGTCTGTTTGAAG | [NM_029767](http://www.ncbi.nlm.nih.gov/nuccore/NM_029767.2) |
| Rps9 low | GAGTCCAGGCGAACAATGAAGGATG |  |
| Slc32a1 up | CGTCGAGGGAGACATTCATT | [NM_009508](http://www.ncbi.nlm.nih.gov/nuccore/NM_009508.2) |
| Slc32a1 low | TGAGGAACAACCCCAGGTAG |  |
| Svopl up | CAGAACCTGCAGCAGAAAGA | [NM_177200](http://www.ncbi.nlm.nih.gov/nuccore/NM_177200.4) |
| Svopl low | CTGGATACCATGTAGCCAAAAA |  |
| Tcfcp2l1 | GTTTTATCCTCAGCACATTAAAAGC | NM_023755 |
| Tcfcp2l1 | GAGACAGCAGCTGACAGTGG |  |
| Uncx up | GGTCGAGTCCCGAGTTCAG | [NM_013702](http://www.ncbi.nlm.nih.gov/nuccore/NM_013702.3) |
| Uncx low | CGATCTCCTCTGGGTCCAT |  |
