# Supplementary material for: Comparison of the Gene Expression Profiles from Normal and Fgfrl1 Deficient Mouse Kidneys Reveals Downstream Targets of Fgfrl1 Signaling
Source: PLoS One. 2012 Mar 14;7(3):e33457. doi: 10.1371/journal.pone.0033457 (PMC3303837; doi:10.1371/journal.pone.0033457)
Supplement: Table S2 — Primers used for WISH. Underlined nucleotides indicate restriction sites used for subcloning. (DOC) [file pone.0033457.s002.doc]

| **Table S2. Primers used for WISH**  Underlined nucleotides indicate restriction sites used for subcloning. | |  |
| --- | --- | --- |
| Primer name | **Sequence (5'→3')** | Accession number |
| Calb1 up | ATGGATCCGACGGAAGTGGTTACCTGGA | NM_009788 |
| Calb1 low | TATCTAGATAAGAGCAAGGTCTGTTCGGTA |  |
| Clec18a up | ATGGATCCCTGAAGGGCTGCAGTACAGA | [NM_181549](http://www.ncbi.nlm.nih.gov/nuccore/NM_181549.3) |
| Clec18a low | TATCTAGATCACTGTCAGTCACCTCGTTG |  |
| Dach1 up | ATGAATTCATCCAGGGTGGAGACATCT | [NM_007826](http://www.ncbi.nlm.nih.gov/nuccore/NM_007826.2) |
| Dach1 low | TATCTAGATCAGGGGTCAGGGAGTCAT |  |
| Dkk1 up | ATGGATCCAGTTGAGGTTCCGCAGTCC | [NM_010051](http://www.ncbi.nlm.nih.gov/nuccore/NM_010051.3) |
| Dkk1 low | TATCTAGAGCTGGCTTGATGGTGATCTT |  |
| Frzb up | ATGGATCCACAGCACCCAGGCTAACG | [NM_011356](http://www.ncbi.nlm.nih.gov/nuccore/NM_011356.4) |
| Frzb low | TATCTAGAGAGCCTTCTACCAAGAGTAACCTG |  |
| Il17rd up | ATGGATCCGGCAGTCACCATTCTTTGGT | [NM_134437](http://www.ncbi.nlm.nih.gov/nuccore/NM_134437.3) |
| Il17rd low | TATCTAGAGCGAACGCAGATATGACAAC |  |
| Krt23 up | ATGGATCCAGAGAACCCAGCCACAGAAA | [NM_033373](http://www.ncbi.nlm.nih.gov/nuccore/NM_033373.1) |
| Krt23 low | TATCTAGAGCAGCACCTTGTGTTCATTG |  |
| Pcp4 up | ATGGATCCAAAAGCCAGGACCAGGGAAG | [NM_008791](http://www.ncbi.nlm.nih.gov/nuccore/NM_008791.2) |
| Pcp4 low | TATCTAGAGGGGCATAAATACTATGGGTTTCT |  |
| Slc32a1 up | ATGGATCCACTCGTATGTGGCCATAGCTAAC | [NM_009508](http://www.ncbi.nlm.nih.gov/nuccore/NM_009508.2) |
| Slc32a1 low | TATCTAGAGCCTTCCTGGAAGAGAGACTTC |  |
| Sp5 up | ATGGATCCCACCTGCCGCCAGTTATC | [NM_022435](http://www.ncbi.nlm.nih.gov/nuccore/NM_022435.2) |
| Sp5 low | TATCTAGAAAGGTGCTGGGAAAGATGTC |  |
| Spry1 up | ATGGATCCTCTTTGTGCCTACCCTGCTT | [NM_011896](http://www.ncbi.nlm.nih.gov/nuccore/NM_011896.2) |
| Spry1 low | TATCTAGATGAAGGGAAGTCCTTCACAGATA |  |
